# Synthesis of 5-(6-hydroxy-7*H*-purine-8-ylthio)- 2-(*N*-hydroxyformamido)pentanoic acid

**DOI:** 10.3762/bjoc.6.93

**Published:** 2010-09-01

**Authors:** Yanmei Zhang, Greg Elliot, Adrian Saldanha, Igor Tsigelny, Dennis Carson, Wolf Wrasidlo

**Affiliations:** 1 Moores Cancer Center, University of California San Diego, La Jolla, CA, 92093, USA

**Keywords:** adenylosuccinate synthetase (AdSS), thiolated nucleoside

## Abstract

We have developed a synthetic route for the preparation of a hybrid bisubstrate small molecule based on a nucleoside. A prototype compound was designed and docked into the catalytic domain of the AdSS enzyme bridging the region between the magnesium center of the protein to the nucleoside region. The synthesis involves coupling a brominated peptide fragment capable of complexing magnesium to a thiolated nucleoside to obtain the hybrid model compound.

## Introduction

**Figure 1:**
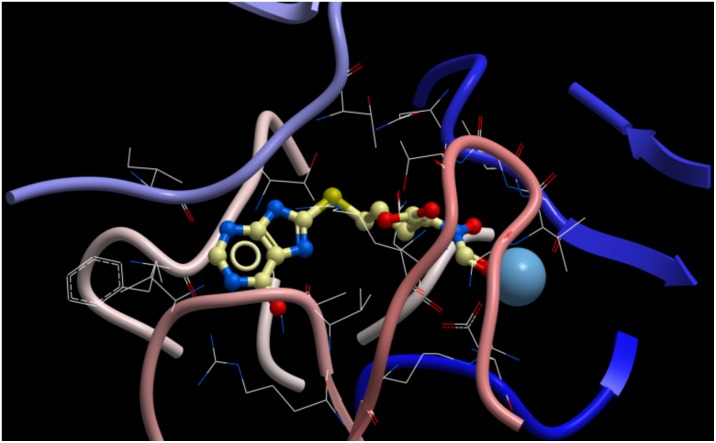
Structure of AdSS catalytic domain from Mg center (light blue) to IMP loop (tan) with compound **13** docked.

During cell proliferation, adenine nucleotides for nucleic acid synthesis are generated through a de novo biosynthetic route, which requires the key enzyme adenylosuccinate synthetase (AdSS). Adenine nucleotides are also recycled by a salvage pathway which requires methylthioadenosine phosphorylase (MTAP). In some cancers this salvage pathway is lost making these cancers hypersensitive to cytotoxic effects of pharmacologic AdSS inhibitors [1]. However, no direct binding inhibitors of human AdSS are known. Recently, the crystal structure of AdSS in the presence of their substrates IMP, GTP and aspartic acid was published [2]. We have used this information in computational models for the design of compounds capable of interfering with the catalytic function of this enzyme [3]. Figure 1 shows the orientation of our model compound within the catalytic domain of AdSS. The synthesis of this hybrid molecule is described below.

## Results and Discussion

**Scheme 1:**
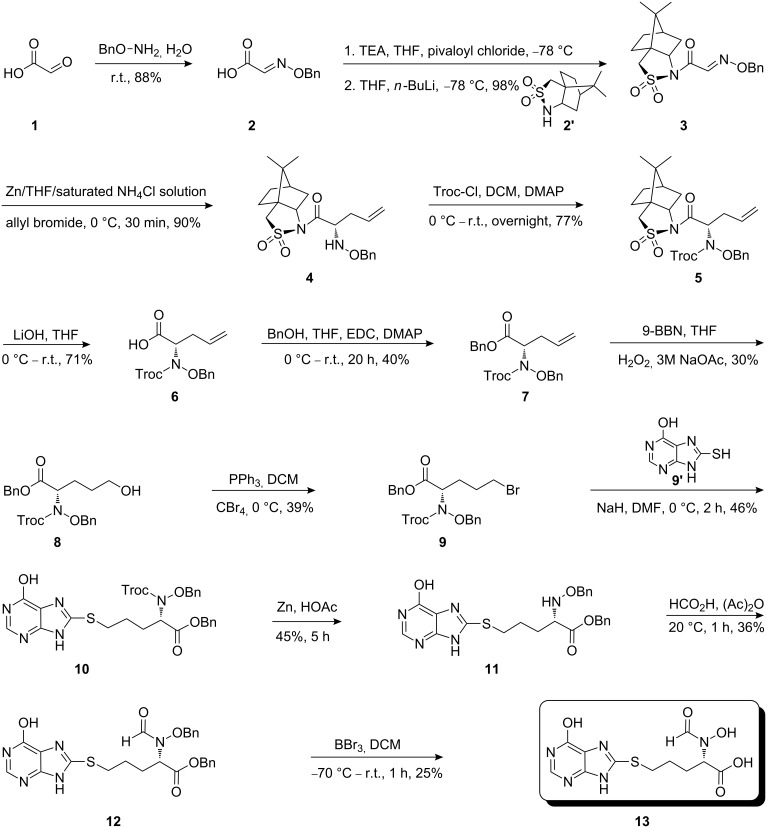
Synthesis of the model compound **13**.

The synthetic pathway to the model compound **13** is outlined in Scheme 1. The reaction of 2-oxoacetic acid (**1**) with *O*-benzylhydroxylamine resulted in benzylhydroxylamino acetic acid **2** in near quantitative yield [4]. The latter compound was converted into the camphorsultam derivative **3**, which was treated with allyl bromide in the presence of powdered zinc in a mixture of THF and aqueous ammonium chloride to produce the allylglycine derivative **4** in excellent yield with high diastereoselectivity [4]. Protection of the amino group as the *N*-trichloroethoxy-carbonyl derivative **5**, followed by cleavage of the chiral amide, esterification and reduction afforded the benzyl ester **8** (via intermediates **6** and **7**) in good overall yield. Treatment of **8** with triphenylphosphine in carbon tetrabromide resulted in the bromide **9** [5–6], which was coupled to mercaptopurine **9’** in the presence of sodium hydride in DMF to yield **10** [7–8] in excellent yield.

Removal of the *N*-Troc group with zinc in acetic acid gave intermediate **11** and subsequent *N*-formylation produced **12**. Finally, debenzylation with boron tribromide afforded the hybrid molecule **13** as a slightly tan powder [9–12]. All compounds were fully characterized and their structures confirmed by ^1^H NMR and mass spectroscopy. The purity of the final product was determined by HPLC and found to be 98%.

Our model compound was docked into the catalytic domain of AdSS as shown in Figure 1. The compound exhibited a close fit with interactions of the peptide part of the structure complexed to the Mg ion of the enzyme and the heterocyclic part located near the IMP loop.

## Experimental

### 

#### Preparation of compound 2


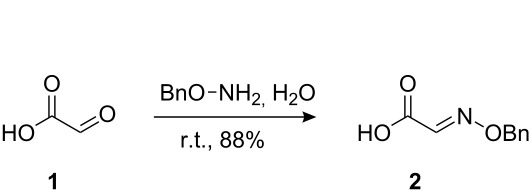


**Compound 2:** Compound **1** (50% aqueous solution) (1.48 g, 0.02 mol) was added dropwise to the solution of *O*-benzylhydroxylamine (1.67 g, 0.014 mol) in 98.0 mL of water at room temperature and the resulting mixture stirred for 4 h. The pH was adjusted to 12 and washed with ethyl acetate. The pH of the aqueous layer was adjusted to pH 1–2 with 2 N HCl and extracted with ethyl acetate. The combined organic layers were dried with sodium sulfate and concentrated in vacuum to give compound **2** (1.57 g, 88%). ^1^H NMR (400 MHz, CDCl_3_): δ 7.54 (s, 1H), 7.34–7.41 (m, 5H), 5.32 (s, 2H). [M + H]^+^: 180.1.

#### Preparation of compound 3


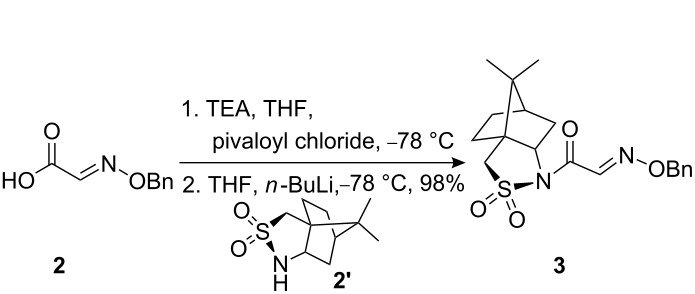


**Compound 3:** Pivaloyl chloride (0.80 mL, 6.49 mmol) was added dropwise to a mixture of compound **2** (1.08 g, 6.04 mmol), Et_3_N (1.2 mL) and THF (30.0 mL) at −78 °C. The mixture was stirred at −78 °C for 10 min then warmed to 0 °C, stirred for 30 min. and then re-cooled to −78 °C. *n*-Butyllithium (2.5 M in hexane, 1.95 mL, 4.87 mmol) was added dropwise to a solution of sultam **2’** (1.0 g, 4.64 mmol) in dry THF (20.0 mL) at −78 °C. The solution was stirred at −78 °C for 10 min, and then transferred, via a cannula, to the solution of the mixed anhydride prepared above. The reaction mixture was stirred at −78 °C for 5 min, and then at 0 °C for 1 h. Saturated ammonium chloride solution (15.0 mL) was added and the mixture extracted three times with ethyl acetate. The combined ethyl acetate extracts were washed successively with water and brine, dried with magnesium sulfate and concentrated under reduced pressure. The residue was recrystallized from hexane–EtOAc to afford the title compound as colorless crystals (1.7 g, 98%). ^1^H NMR (300 MHz, CDCl_3_): δ 8.13 (s, 1H), 7.19–7.29 (m, 5H), 5.23 (s, 2H), 4.03 (t, *J* = 7.2 Hz, 1H), 3.34–3.47 (m, 2H), 1.96–2.08 (m, 2H), 1.79–1.90 (m, 3H), 1.22–1.37 (m, 2H), 1.07 (s, 3H), 0.89 (s, 3H). [M + H]^+^: 377.4.

#### Preparation of compound 4


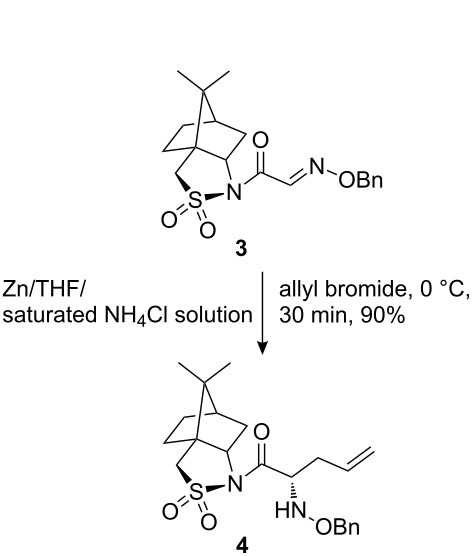


**Compound 4:** To a vigorously stirred solution of compound **3** (1.76 g) in THF (9.0 mL) and saturated ammonium chloride (9.0 mL) at 0 °C, was added allyl bromide (671.0 µL) followed by zinc powder (0.62 g). The zinc dust was added portionwise at such a rate that one portion was allowed to react before adding the next. The mixture was stirred at 0 °C for 30 min, then partitioned between ethyl acetate and water. The aqueous layer was re-extracted with ethyl acetate. The combined ethyl acetate extracts were washed with brine, dried with MgSO_4_ and concentrated. The residue was recrystallized from hexane–EtOAc to afford the desired product as colorless crystals (1.0 g, 90%). ^1^H NMR (300 MHz, CDCl_3_): δ 7.26–7.37 (m, 5H), 5.69–5.78 (m, 1H), 5.02–5.10 (m, 2H), 4.65–4.75 (m, 2H), 4.45–4.49 (m, 1H), 3.95–3.99 (m, 1H), 3.44–3.54 (m, 2H), 2.27–2.40 (m, 2H), 2.04–2.08 (m, 2H), 1.86–1.92 (m, 3H), 1.15(s, 3H), 0.94(s, 3H). [M + H]^+^: 419.5.

#### Preparation of compound 5


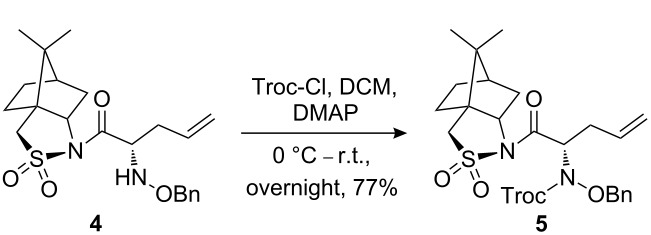


**Compound 5:** To a mixture of compound **4** (1.9 g, 4.54 mmol), DMAP (20.0 mg) and dichloromethane (16.0 mL) at 0 °C, was added pyridine (0.43 g, 5.44 mmol) followed by the dropwise addition of trichloroethoxy chloroformate (1.17 g, 5.52 mmol). The mixture was stirred at 0 °C for 2 h, then warmed to room temperature and stirred at r.t. overnight. The reaction mixture was diluted with DCM and quenched with saturated ammonium chloride. The organic layer was washed successively with water and brine, dried with sodium sulfate and concentrated. The residue was purified by chromatography with DCM–MeOH (120:1) as eluent to yield the title compound **5** as a white foam (2.1 g, 77%). ^1^H NMR (400 MHz, CDCl_3_): δ 7.40–7.43 (m, 2H), 7.26–7.30 (m, 3H), 5.71–5.79 (m, 1H), 4.67–5.31 (m, 7H), 3.83–3.89 (m, 1H), 3.39–3.46 (m, 2H), 2.67–2.86 (m, 2H), 2.01–2.07 (m, 2H), 1.79–1.90 (m, 3H), 1.24–1.39 (m, 2H), 1.08 (s, 3H), 0.91 (s, 3H). [M + H]^+^: 594.9.

#### Preparation of compound 6


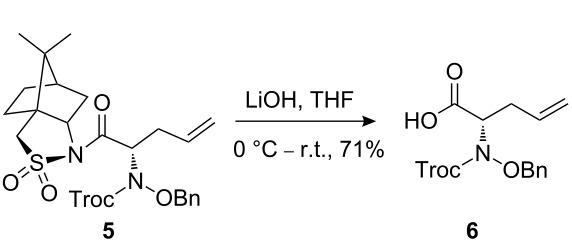


**Compound 6:** A 1 N lithium hydroxide solution (5.25 mL, 5.25 mmol) was added dropwise to a solution of compound **5** (2.1 g, 3.54 mmol) in THF (22.0 mL) at 0 °C. The mixture was stirred at 0 °C for 1 h, then at room temperature for 5 h. The reaction mixture was diluted with ethyl acetate (25.0 mL) and water (25.0 mL). The aqueous phase was extracted with ethyl acetate (2 × 25 mL) to remove camphor sultam, then acidified with 2 N HCl (until pH 2). The cloudy aqueous solution was then extracted with ethyl acetate (3 × 25 mL). The combined ethyl acetate solution was dried with sodium sulfate and concentrated. The residue was purified by column chromatography with DCM–MeOH–AcOH (120:20:1) as eluent to give the desired product **6** as a colorless oil (1.0 g, 71%). ^1^H NMR (400 MHz, CDCl_3_): δ 7.34–7.42 (m, 5H), 5.77–5.86 (m, 1H), 4.76–5.30 (m, 6H), 2.76–2.82 (m, 2H). [M + H]^+^: 397.6.

#### Preparation of compound 7


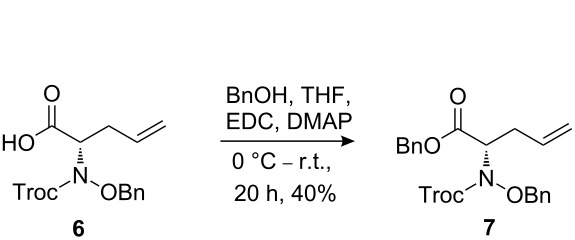


**Compound 7:** To a solution of the acid **6** (491.0 mg, 1.24 mmol) in anhydrous THF (20.0 mL) at 0 °C, was added 1-(3-dimethylaminopropyl)-3-ethylcarbodiimide hydrochloride (EDC, 475.0 mg, 2.48 mmol) followed by DMAP (302.0 mg, 2.48 mmol). After stirring at 0 °C for 30 min, benzyl alcohol (402.0 mg, 3.72 mmol) was added. The reaction mixture was stirred at 0 °C for 1 h, then warmed to room temperature and stirred for 18 h. The mixture was diluted with ethyl acetate, and washed successively with water and brine, dried (MgSO_4_) and concentrated. The residue was purified by column chromatography with hexane–EtOAc (4:1) as eluent to give the desired product as a colorless oil (240.0 mg, 40%). ^1^H NMR (300 MHz, CDCl_3_): δ 7.32–7.47 (m, 10H), 5.76–5.89 (m, 1H), 4.73–5.40 (m, 9H), 2.70–2.86 (m, 2H). [M + H]^+^: 487.7.

#### Preparation of compound 8


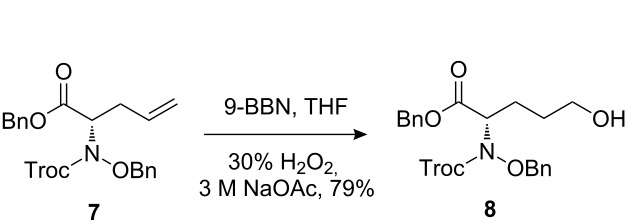


**Compound 8:** A solution of 9-BBN (0.5 M in THF, 4.84 mL, 2.4 mmol) was added slowly to a solution of compound **7** (587.0 mg, 1.2 mmol) in anhydrous THF (4.0 mL) at 0 °C. The reaction mixture was warmed to room temperature. After stirring at this temperature for 23 h, the reaction mixture was cooled to 0 °C. 3 M NaOAc (3.43 mL) and 30% H_2_O_2_ (1.71 mL) were added successively. The mixture was heated and stirred at 50 °C for 4 h. After cooling to room temperature, the aqueous layer was extracted with ethyl acetate. The ethyl acetate extracts were washed in turn with water and brine, dried with sodium sulfate and concentrated. The residue was purified by silica gel column chromatography. The column was eluted first with hexane–EtOAc (10:1) and then with hexane–EtOAc–Et_3_N (80:40:1) to give the desired product as a colorless oil (483.0 mg, 79%). ^1^H NMR (300 MHz, CDCl_3_): δ 7.22–7.34 (m, 10H), 4.91–5.19 (m, 4H), 4.62–4.76 (m, 3H), 3.54–3.62 (m, 2H), 1.91–2.15 (m, 2H), 1.36–1.70 (m, 2H). [M + H]^+^: 505.7.

#### Preparation of compound 9


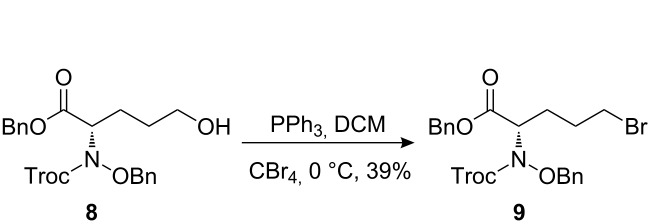


**Compound 9:** To a mixture of compound **8** (0.48 g, 1 equiv), CBr_4_ (0.48 g, 1.5 equiv) and DCM (9.0 mL) at 0 °C, was added a solution of PPh_3_ (0.38 g, 1.5 equiv) and DCM (4.0 mL). The resulting mixture was stirred at room temperature until the starting materials disappeared, the solvent was removed in vacuum and the residue was purified by silica gel column chromatography to give the product (0.22 g, 39%). ^1^H NMR (400 MHz, CDCl_3_): δ 7.23–7.29 (m, 10H), 5.04–5.16 (m, 2H), 4.88–5.01 (m, 2H), 4.58–4.77 (m, 3H), 3.26–3.31 (m, 2H), 1.86–2.12 (m, 4H). [M + H]^+^: 568.6.

#### Preparation of compound 10


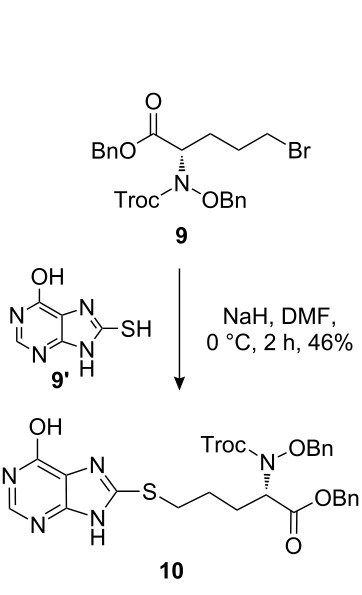


**Compound 10:** To a solution of compound **9’** (50.0 mg, 1 equiv) in DMF (1.0 mL) at 0 °C, was added sodium hydride (11.9 mg, 1.1 equiv). After stirring at 0 °C for 2 h, a solution of compound **9** (169.0 mg, 1 equiv) in DMF (0.3 mL) was added within 1 min. The resulting mixture was stirred for 25 min, then quenched with ammonium chloride The mixture was extracted with ethyl acetate, dried, concentrated and purified by column chromatography on silica gel to yield compound **10** (90.0 mg, 46%). ^1^H NMR (400 MHz, CDCl_3_): δ 13.30 (brs, 1H), 12.20 (s, 1H), 7.91 (s, 1H), 7.27–7.36 (m, 10H), 5.09–5.17 (m, 2H), 4.80–4.96 (m, 5H), 3.20–3.28 (m, 2H), 2.01–2.19 (m, 2H), 1.75–1.88 (m, 2H). [M + H]^+^: 655.9.

#### Preparation of compound 11


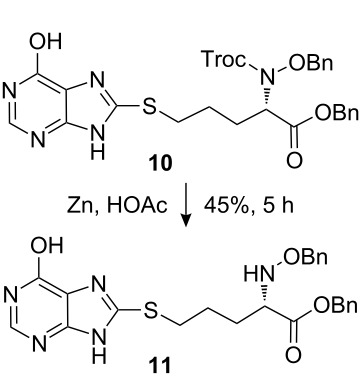


**Compound 11:** Compound **10** (90.0 mg) was dissolved in acetic acid (1.0 mL) and treated with zinc dust (160.0 mg). The mixture was stirred at room temperature for 5 h, diluted with ethyl acetate, and filtered through a Celite pad. The ethyl acetate solution was washed successively with saturated potassium carbonate, water and brine, dried with sodium sulfate and concentrated. The residue was purified by column chromatography with DCM–MeOH (95:5) as eluent to give the crude product. The crude product was used in the next step without further purification (30.0 mg, 45%). [M + H]^+^: 480.5.

#### Preparation of compound 12


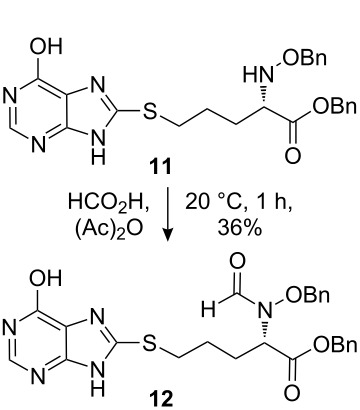


**Compound 12:** To a solution of compound **11** (26.0 mg) in formic acid (96%, 800.0 µL), was added acetic anhydride (15.0 µL). The mixture was stirred at 20 °C for 1 h. After pouring into iced-water, the aqueous layer was extracted with ethyl acetate. The combined ethyl acetate phase was washed successively with potassium carbonate aqueous solution and brine, dried over sodium sulfate, concentrated, and the residue purified with preparative TLC with DCM–MeOH (97:3) as eluent (developed 5 times) to give the desired product **12** as a syrup (10.0 mg, 36%). ^1^H NMR (400 MHz, MeOD): δ 8.12 (s, 1H), 7.83 (s, 1H), 7.20–7.48 (m, 10H), 5.09 (s, 2H), 4.49 (s, 2H), 2.06–2.07 (m, 2H), 1.68–1.77 (m, 2H), 1.12–1.27 (m, 2H). [M + H]^+^: 507.5.

#### Preparation of compound 13


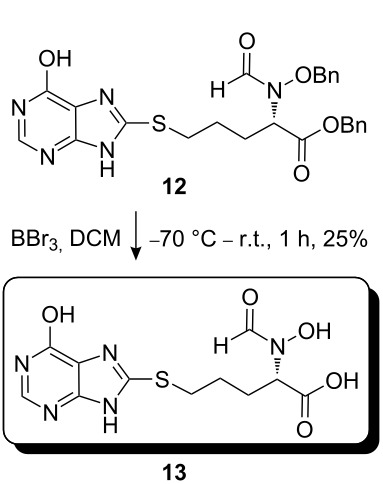


**Compound 13:** To a mixture of compound **12** (30.0 mg) and DCM (10.0 mL) at −70 ^o^C, was added a solution of BBr_3_ (130.0 mg) in DCM (1.0 mL). The mixture was allowed to warm to room temperature, stirred for one additional hour and then poured into iced-water. The aqueous layer was washed with dichloromethane and adjusted to pH 3–4. The remaining aqueous solution was concentrated in vacuum, and the residue was washed with THF. The THF layer was concentrated and purified by preparative HPLC to give the product (5.0 mg, 25%). ^1^H NMR (400 MHz, CDCl_3_): δ 12.80 (brs, 1H), 12.21 (s, 1H), 10.06 (s, 0.5H), 9.81 (brs, 0.5H), 8.36 (s, 0.5H), 8.02 (s, 0.5H), 7.93 (s, 1H), 4.74–4.78 (m, 0.5H), 4.49–4.52 (m, 0.5H), 3.22–3.27 (m, 2H), 1.88–1.95 (m, 2H), 1.62–1.70 (m, 2H). [M + H]^+^: 328.3.
